# Regarding Iodixanol for Pediatric CTA. Comment on Pop, M. Cardiothoracic CTA in Infants Referred for Aortic Arch Evaluation—Retrospective Comparison of Iomeprol 350, Ioversol 350, Iopromide 370 and Iodixanol 320. *Children* 2021, *8*, 949

**DOI:** 10.3390/children9050696

**Published:** 2022-05-10

**Authors:** Ulf Nyman, Peter Aspelin

**Affiliations:** 1 Department of Translational Medicine, Division of Medical Radiology, University of Lund, SE-20502 Malmö, Sweden; 2 Department of Clinical Science, Intervention and Technology (CLINTEC), Karolinska Institute and Karolinska University Hospital, SE-14186 Stockholm, Sweden; peter.aspelin@ki.se

With interest, we read the publication ‘Cardiothoracic CTA in Infants Referred for Aortic Arch Evaluation—Retrospective Comparison of Iomeprol 350, Ioversol 350, Iopromide 370 and Iodixanol 320′ by Pop [1].

We are surprised to see that the authors report a significantly lower enhancement rate after iso-osmolar iodixanol 320 (Visipaque™) as compared to the low-osmolar CM. As most radiologists will know, enhancement in CT angiography (CTA) is not defined primarily by the concentration of administered iodine but by the iodine delivery rate (IDR) in terms of mg I/kg per second [2], which is of particular importance in children, where body weight will vary substantially. In the present study, injection flow was adapted to body habitus (0.5–1.6 mL/s) to account for differences in body weight. However, from the methods section, it is not clear whether CM was administered in terms of mg I/kg at constant injection time, which is crucial for the comparison of CM with different concentrations [3]. Hence, the mean IDR in mg I/kg/s shown in Table 2, which was surprisingly high but lowest for iodixanol (*p* = NS), does not allow for a true comparison of the CM groups. Furthermore, any differences in cardiac output, another crucial parameter regarding vascular CM enhancement, have not been accounted for [4].

Pop et al. [1]. switch between reporting mean and median values. They report the mean of enhancement, which may be wrong in this very small sample, just because we do not know if there was a normal distribution. In contrast, they report median age, which was 111 days (nearly 4 months) in the iodixanol group but 7–10 days in the other groups (*p* = 0.056). Median weights and heights were much more similar between the groups. Hence, we wonder whether these children were born severely premature or had significant co-morbidities affecting weight and height which could also impact cardiac output and hence levels of contrast media enhancement in the aorta.

Iso-osmolar iodixanol is also considered particularly suitable in children compared to low-osmolar CM that are 2–3 times more hyperosmolar compared to plasma at concentrations ranging from 300 to 400 mg I/mL. This is pointed out by the American College of Radiology in their 2020 contrast media guideline: *‘CM osmolality is of particular importance in neonates and small children. Hyperosmolar CM may result in expanding blood volume and if fluid shift is large, cardiac failure and pulmonary edema can result’*.

Pop postulated that differences in the intrinsic flow and vascular distribution of CM, resulting in lower enhancement of iodixanol, may in part be due to its high viscosity at room temperature. In fact, blood viscosity and intrinsic blood flow is more hampered by the hypertonicity of a CM, causing the rigidification of red blood cells, than its viscosity, which is why an iso-osmolar CM is preferred. Iodixanol’s high viscosity can be reduced by about 50% upon prewarming the contrast to 37 °C. However, we do not know whether the CM was pre-warmed in the study by Pop, nor did we see any mention of injection site or catheter/needle sizes being used that may reflect anticipated differences. Another option would be to use iodixanol 270 mg I/mL with less than half the viscosity of 320 mg I/mL. Furthermore, the densities of the pulmonary artery in Pop’s study, ranging from about 680 to 320 HU, indicate a blood CM concentration of 8 to 17 mg I/mL at 80 kV (40 HU per mg I/mL at 80 kV [5]) with negligible viscosity.

Considering the small sample size of this retrospective comparison (n = 11–13 in each CM group) and the scientific and analytical weaknesses described above, the authors’ conclusion that ‘in CTA of infants suspected of aortic arch hypoplasia/coarctation, iodixanol 320 provided up to 40% less enhancement of the great vessels when compared to iomeprol 350, ioversol 350 and iopromide 370′ appears unjustified. Iodixanol is being used in various indications, including CT, especially in vulnerable patients with impaired kidney function, and is considered particularly suitable in children. It is the standard CM in Sweden for these examinations in children.

## Data Availability

Not applicable.
